# Ten Year Outcome of Anti‐Thyroid Drug Treatment for First Episode Graves' Thyrotoxicosis: The Predictive Importance of TRAb

**DOI:** 10.1111/cen.70003

**Published:** 2025-07-08

**Authors:** Nyo Nyo Z. Tun, Nicola N. Zammitt, Mark W. J. Strachan, Jonathan R. Seckl, Fraser W. Gibb

**Affiliations:** ^1^ Edinburgh Centre for Endocrinology & Diabetes Edinburgh UK; ^2^ University/BHF Centre for Cardiovascular Science University of Edinburgh Edinburgh UK

**Keywords:** Graves', hyperthyroidism, thionamide, thyrotoxicosis, TRAb

## Abstract

**Objective:**

To establish the risk and time course of recurrent thyrotoxicosis following withdrawal of anti‐thyroid drugs (ATD) and risk factors for recurrence.

**Design:**

Single‐centre retrospective study.

**Methods:**

Two hundred and ninety people with a first episode of Graves' thyrotoxicosis, who completed a course of ATD, were included. Clinical and biochemical parameters associated with recurrence risk were assessed over a 10‐year period.

**Results:**

Recurrence occurred in 54% of individuals over a 10‐year period, with 73% occurring within 2 years. Younger age (41 years [33–51] vs. 47 [39–56], *p* = 0.011), higher TSH receptor antibody (TRAb) at diagnosis (8.8 IU/L [4.9−17.2] versus 6.0 [4.1−9.9], *p* = 0.002), higher TRAb at cessation of ATD (1.3 [<0.9–2.3] vs. 1.0 [<0.9–1.3], *p* < 0.001), longer time to normalisation of TSH (6 months [3–9] vs. 4 [2–7], *p* 0.013) and longer time to normalisation of fT4 (2 months [1−3] vs. 1 [1−2], *p* = 0.001) were all associated with relapse within 10 years. Recurrence within 10 years occurred in 74% of individuals with TRAb > 12 IU/L at diagnosis but only 44% of those with TRAb < 5 IU/L at diagnosis (*p* = 0.001). TRAb (at diagnosis and cessation) and age were independently associated with relapse in multivariate analysis.

**Conclusions:**

Most recurrent thyrotoxicosis occurs within the first few years after ATD withdrawal. TRAb concentration, at diagnosis and cessation of ATD, is a useful predictor of recurrence risk and can be used to inform decisions on the optimal approach to primary therapy.

## 1 Introduction

Anti‐thyroid drugs (ATDs) are safe and effective in promptly restoring euthyroidism in people with Graves' thyrotoxicosis [1]. Treatment is typically administered for a 12–18 month period but recurrence following withdrawal is common; approximately 50% in Europe and up to 80% in North America [1]. TSH receptor antibodies (TRAb) are elevated at diagnosis of thyrotoxicosis in approximately 97% of people with Graves' disease [2]. In addition to their role in establishing the diagnosis of Graves' disease, TRAb has been associated with predicting the risk of recurrence following withdrawal of ATD, as well as the risk of radioiodine treatment failure [3, 4]. Other features associated with increased risk for recurrence include younger age [5], large goitre size [6], male sex [5], orbitopathy [6] and cigarette smoking [6], although there is significant variability in the published evidence. Previous studies have not necessarily been consistent in assessing people who have completed a physician directed course of ATD (with planned cessation) and most studies have been limited by extremely short follow‐up (typically 1–2 years). This study sought to report the natural history of recurrence over a 10‐year period and to establish risk factors for recurrent thyrotoxicosis following ATD withdrawal.

## 2 Materials and Methods

### 2.1 Patients

Two hundred and ninety consecutive patients were identified with a first presentation of Graves' thyrotoxicosis (with accompanying TRAb levels) who completed a physician‐directed course of ATD therapy (2006–2011). Serial thyroid function tests were recorded for at least 10 years after drug withdrawal.

### 2.2 Assays

Free thyroxine (reference range 9–21 pM), total triiodothyronine (0.9–2.4 nM) and TSH (0.2–4.5 IU/L) measurements were performed on an Abbott Architect Immunoassay system (Abbott Diagnostics, Maidenhead, UK) from 2007 onwards. Before this, assays were performed on either Advia Centaur (Siemens Healthcare Diagnostics, Surrey, UK) or the Vitro ECi platform (Ortho Clinical Diagnostics, High Wycombe, UK) across the two sites comprising our centre. Regression equations were used to correct non‐architect derived values reported in this study (Dr. G. Beckett and Ms. C, Shearing, Clinical Biochemistry Department, NHS Lothian, personal communication). TRAbs were measured using BRAHMS LUMItest TRAK assay (BRAHMS Diagnostica, Berlin, Germany) until 2008 when replaced by a Roche assay on the Cobas E411 analytical platform (Roche Diagnostics, West Sussex, UK) [7]. No significant differences were noted in results between the two TRAb assays [8]. The functional sensitivity of the TRAb assays was 0.9 IU/L, with normal TRAb levels < 1.5 IU/L. Anti‐TPO antibodies were measured using Orgentec enzyme‐linked immunosorbent assay (Mainz, Germany). The reference range for anti‐TPO antibodies is 0–150 IU/L.

### 2.3 Biochemical Parameters

High serum fT4 or TT3, suppressed TSH, and a positive TRAb titre established a biochemical diagnosis of Graves' thyrotoxicosis. All follow‐up thyroid function tests were recorded and time to normalisation of TSH and fT4 was calculated. As a real‐world assessment, there were no fixed follow‐up intervals but monitoring was typically performed every 6–8 weeks in the early phase of therapy. TRAb concentration at baseline was available in all individuals and TRAb before cessation of ATD was available in 144 (75% of those included in the analysis of 10‐year outcomes). After ATD withdrawal, all subsequent thyroid function tests were recorded. Anti‐TPO antibodies were recorded in 104 patients at diagnosis (55%). Time to relapse, after ATD withdrawal, was defined by the first date of biochemical thyrotoxicosis.

### 2.4 Therapy

Standard practice was to prescribe an 18‐month tapering course of carbimazole, with a typical starting dose of 40 mg. Propylthiouracil (PTU) was used in the context of planned pregnancy or carbimazole intolerance. Block and replace regimens were not used in this cohort.

### 2.5 Statistics

The primary outcome measure was recurrent thyrotoxicosis following ATD withdrawal. For statistical analysis, values below assay limit of detection were recorded as zero and values above the upper limit for reporting are assigned the highest reported value. TRAb categories were established pragmatically for numerical balance between groups and have been used previously [3]. Continuous unpaired data were presented as median (IQR) and compared by Wilcoxon rank‐sum test. Categorical data were compared by *χ* ^2^ test. Relapse data were presented using Kaplan−Meier survival plots and groups are compared by log‐rank test. Cox proportional hazard models were used to establish independent risk factors for recurrence. *p* values < 0.05 were considered statistically significant. Statistical analyses were performed using R Studio (version 2023.12.1).

## 3 Results

### 3.1 Baseline Characteristics

**Figure 1:**
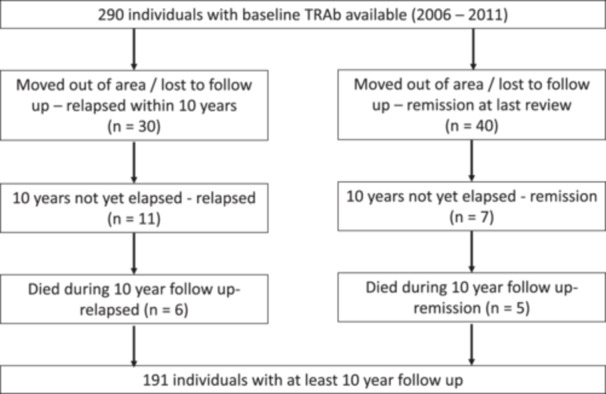
Description of cohort and reasons for exclusion from final analysis.

Of 290 people with baseline TRAb data available, 191 had completed 10‐year follow‐up and their outcomes are reported. Reasons for non‐inclusion are reported in Figure 1. There were no differences of note, in terms of demographics or presenting biochemistry, between those with 10‐year follow‐up data and those not included in the final analysis (Table 1). 80% of patients were female. At completion of ATD therapy, 175/191 (92%) were on carbimazole and 16/191 (8%) were on PTU. The median duration of ATD therapy was 17 months (16–19).

**Table 1:** Baseline characteristics of the 10‐year follow‐up cohort and comparison with those not included in the final analysis.

	10‐year cohort (*n* = 191)	Not included (*n* = 99)	*p*
Age at diagnosis (years)	44 (35–54)	41 (31–52)	0.191
Gender	Female 153 (80%) Male 38 (20%)	Female 72 (73%) Male 27 (27%)	0.201
Free T4 (pM) at diagnosis	32 (25–39)	31 (24–38)	0.546
Total T3 (nM) at diagnosis	4.6 (3.3–5.8)	4.4 (3.1–6.1)	0.996
TRAb (IU/L) at diagnosis	7.5 (4.4–12.9)	7.6 (4.5–11.9)	0.982
Anti‐TPO (IU/L) at diagnosis	88 (25–297)	113 (44–382)	0.328
Duration of therapy (months)	17 (16–19)	18 (17–21)	0.055
TRAb at cessation (IU/L)	1.1 (<0.9–1.7)	1.0 (<0.9–1.5)	0.301
Duration of normal TSH (months)	12 (9–15)	12 (9–15)	0.599
Duration of normal fT4 (months)	16 (14–18)	16 (14–19)	0.208
Time to normalisation of TSH (months)	4 (3 – 6)	6 (3–9)	0.079
Time to normalisation of fT4 (months)	1 (1–2)	2 (1–2)	0.036

### 3.2 Relapse and Outcomes

Recurrent thyrotoxicosis occurred in 103/191 people within 10 years (54%). 54% of all relapses (56/103) occurred within 1 year, 72% within 2 years (74/103) and 93% within 5 years (96/103). Using the final relapse status of those excluded from the main analysis (due to death or loss to follow‐up), the recurrence rate was broadly similar at 51% (139/272). Median follow‐up duration was 141 months (128–160) and the status of all individuals is reported in Table 2. Only a single further case of recurrent thyrotoxicosis was observed during extended follow‐up. There was no significant difference in the likelihood of not being on therapy at final follow‐up (ATD or thyroid hormone replacement) based on TRAb category at diagnosis (high: 46% medium: 53% low: 54%, *p* = 0.660). Higher TRAb at cessation of therapy, longer duration of therapy and longer time to normalisation of fT4 were associated with higher likelihood of being on either ATD or levothyroxine at final follow‐up (supporting Information S1: Table).

**Table 2:** Outcomes at final follow‐up (median 141 months). Long‐term ATD refers to planned indefinite therapy with no plan to discontinue.

	Total (*n* = 191)	Remission at 10 years (*n* = 88)	Relapse within 10 years (*n* = 103)
Deceased	8 (4.2%)	5 (5.7%)	3 (2.9%)
Spontaneous hypothyroidism (on levothyroxine)	7 (3.7%)	5 (5.7%)	2 (1.9%)
Long‐term ATD	41 (22%)	1 (2.4%)	40 (39%)
No current therapy	94 (49%)	77 (82%)	17 (17%)
Radioiodine	31 (16%)	0 (0%)	31 (30%)
Thyroidectomy	10 (5.2%)	0 (0%)	10 (10%)

### 3.3 Predictive Factors for Recurrent Thyrotoxicosis

**Figure 2:**
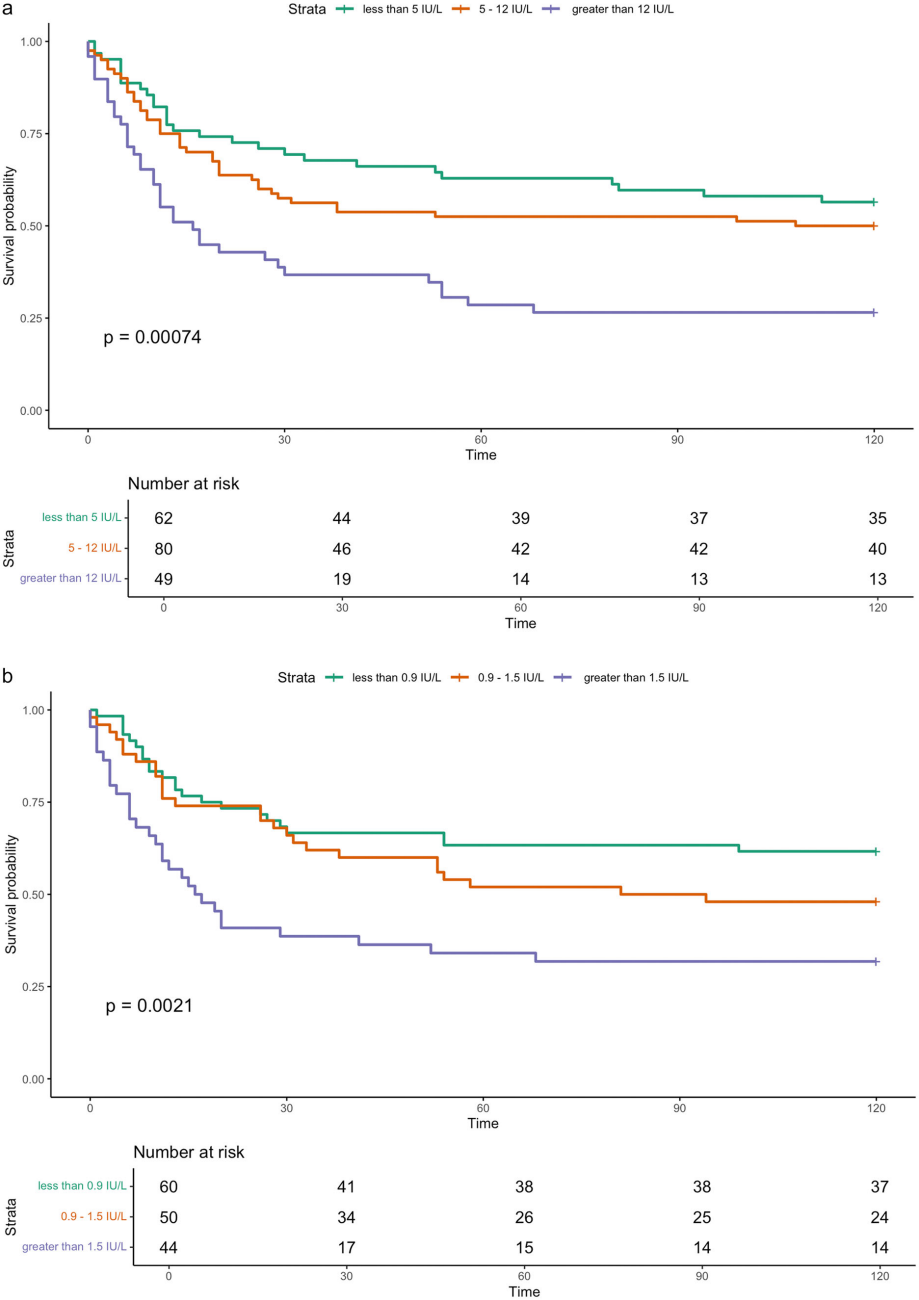
Influence of TRAb category at diagnosis (a) and cessation of ATD (b) upon recurrent thyrotoxicosis.

A comparison of demographic and clinical features between those in remission and those with recurrent thyrotoxicosis within 10 years is presented in Table 3. Younger age, longer duration of ATD treatment, higher TRAb concentration (at diagnosis and cessation of ATD) and longer duration to normalisation of fT4 and TSH were all associated with increased risk of recurrent thyrotoxicosis. Sex, thyroid hormone concentration at diagnosis, anti‐TPO antibody concentration and duration of normal fT4 and TSH during ATD therapy were not associated with recurrence risk. TRAb category both at diagnosis and cessation was significantly associated with the risk of recurrence at 5 and 10 years (Table 4). Survival curves demonstrating the association between TRAb category and recurrence over a 10‐year period are presented in Figure 2. Cox proportional hazard analysis identified the following as independent predictors of relapse: TRAb at diagnosis greater than 12 IU/L (HR 2.4 [95% CI 1.3–4.4], *p* 0.006), TRAb at cessation between 0.9 and 1.5 IU/L (HR 1.8 [95% CI 1.0–3.3], *p* = 0.044), TRAb at cessation greater than 1.5 IU/L (HR 2.3 [95% CI 1.3–4.0], *p* = 0.006) and younger age (HR 0.97 per year [95% CI 0.95–0.99], *p* = 0.004). TRAb at diagnosis between 5 and 12 (HR 1.2 [95% CI 0.7–2.2], *p* = 0.495) and time to normalisation of TSH (HR 1.04 per month [95% CI 0.99–1.09], *p* = 0.088) were not independently associated with recurrence risk in this model.

**Table 3:** Comparison of clinical and biochemical features of those in remission at 10 years versus those with recurrent thyrotoxicosis.

	Remission at 10 years (*n* = 88)	Relapse at 10 years (*n* = 103)	*p*
Age at diagnosis (years)	47 (39–56)	41 (33–51)	0.011
Gender	Female 67 (44%) Male 21 (55%)	Female 86 (56%) Male 17 (45%)	0.277
Free T4 (pM) at diagnosis	31 (24–36)	32 (25–41)	0.125
Total T3 (nM) at diagnosis	4.6 (3.4–5.3)	4.9 (3.3–6.0)	0.324
TRAb (IU/L) at diagnosis	6.0 (4.1–9.9)	8.8 (4.9–17.2)	0.002
Anti‐TPO (IU/L) at diagnosis	69 (25–236)	158 (26–412)	0.148
Duration of therapy (months)	17 (16–18)	18 (16–20)	0.028
TRAb at cessation (IU/L)	1.0 (<0.9–1.3)	1.3 (<0.9–2.3)	<0.001
Duration of normal TSH (months)	13 (9–15)	11 (9–15)	0.554
Duration of normal fT4 (months)	16 (14–18)	16 (14–18)	0.549
Time to normalisation of TSH (months)	4 (2–7)	6 (3–9)	0.013
Time to normalisation of fT4 (months)	1 (1–2)	2 (1–3)	0.001

**Table 4:** 5‐ and 10‐year recurrence rates by TRAb category (5‐year outcomes: *p* = 0.001 for initial TRAb category and *p* = 0.013 for cessation TRAb category. 10‐year outcomes: *p* = 0.005 for initial TRAb category and *p* = 0.011 for cessation TRAb category).

	5‐Year recurrence	10‐Year recurrence
Total	96/191 (50%)	103/191 (54%)
Initial TRAb high	35/49 (71%)	36/49 (74%)
Initial TRAb intermediate	38/80 (48%)	40/80 (50%)
Initial TRAb low	23/39 (37%)	27/62 (44%)
Cessation TRAb high	29/44 (66%)	30/44 (68%)
Cessation TRAb intermediate	24/40 (48%)	26/50 (52%)
Cessation TRAb low	22/60 (37%)	23/60 (38%)

### 3.4 Prognostic Performance

With respect to predicting the risk of recurrence at 10 years, belonging to the intermediate or high diagnosis TRAb category had a sensitivity of 74% and specificity of 40%. When considering only those in the high TRAb category at diagnosis, sensitivity was 35% and specificity was 85%. At cessation of therapy, detectable TRAb had 60% sensitivity for predicting recurrence (62% specificity).

## 4 Discussion

We have shown that TRAb (both at diagnosis and at ATD withdrawal) remains a predictor of outcome at 10 years of follow‐up. Furthermore, the vast majority of recurrent thyrotoxicosis occurs within 2 years of ATD withdrawal and recurrent thyrotoxicosis after 5 years is uncommon. After almost 12 years of follow‐up, around half of individuals were not on any therapy (ATD or thyroid hormone replacement) and this was not significantly different between TRAb category at diagnosis or cessation of therapy; this is likely to be perceived as an important outcome in people living with Graves' disease.

Observational data suggest that recurrent thyrotoxicosis may have adverse effects upon mortality [9], especially in older individuals [10]. Older individuals are also much less likely to present with classical symptoms of thyrotoxicosis [11]. Despite the association between TRAb and recurrence risk, it is not sufficiently sensitive to permit ATD discontinuation in individuals who would be considered at high clinical risk in the context of recurrent disease (e.g., a 20% risk of recurrence may be unacceptable in an older, high‐risk individual, where an 80% risk may be tolerated in a young, otherwise healthy, person). There are no current methods of risk stratification which adequately classify an individual's risk of thyrotoxicosis [6]; even models incorporating TRAb, age and goitre size perform far from perfectly [12]. Therefore, in people considered to be at higher cardiovascular risk, it may be preferable to consider either long‐term, low‐dose ATD or definitive therapy.

In the United Kingdom, 2019 NICE guidelines [13] suggest radioactive iodine is offered as first‐line definitive therapy but ATDs should be considered where remission is likely to be achieved. However, despite associations between several clinical and demographic factors, it remains extremely difficult, at an individual patient level, to adequately define the risk of recurrence. Furthermore, as our data suggest, more than half of those who complete an ATD course will not require any thyroid‐related therapy at long‐term follow‐up and this is an outcome which is highly valued by patients [14]. Although definitive when successful, radioiodine has an appreciable failure rate and this appears to be greatest in those with highest TRAb levels at diagnosis (precisely the group where early radioiodine may be favoured) [4]. Published data on outcomes with long‐term carbimazole are limited but this approach has been reported to achieve similar thyroid function results, in the context of a virtual thyrotoxicosis clinic, when compared to definitive therapies [15]. An observational study in Italy suggested lower rates of recurrence with persistent ATD therapy, although this effect appeared limited to those over the age of 35 years [16]. The potential efficacy of longer‐term ATD therapy, and the observation that those with highest TRAb concentration will typically relapse within 2 years, suggests an approach of continuing ATD until TRAb is undetectable (or perhaps up to 5 years when recurrence rates drop substantially); although this would require confirmation in a randomised controlled trial.

In contrast to other reports [6], we did not find thyroid hormone levels at presentation to be significantly associated with recurrence risk. Relatively straightforward (and easily measured) metrics such as time to normalisation of TSH and fT4 were associated with recurrence risk but not independently of TRAb. We did not have reliable access to metrics which have previously been associated with recurrence risk such as goitre size [5], ophtlamopathy [17] and smoking status [6]. Incorporating multiple features into a risk score improves prognostic performance [12] but arguably not decisively enough to influence management beyond consideration of TRAb alone. More sophisticated scoring systems which incorporate HLA subtypes appear to have superior prognostic performance but these have not, to date, been incorporated into routine clinical practice [12]. This study specifically reports outcomes in people who have completed a course of ATD and, to broaden generalisability, it would be of value to assess long‐term outcomes in a cohort including all individuals commencing ATD, not just those deemed suitable for discontinuation at 12–18 months. This was an evaluation of a single centre's outcomes which raises questions of generalisability but outcomes are, in general, broadly consistent with the current literature [1].

The key strength of this study is robust assessment of thyroid outcomes over a long period of follow‐up. The information presented is likely to be relevant to healthcare professionals and patients when considering treatment options, including predicted time course of recurrence and chances of avoiding long‐term medication.

## 5 Conclusions

Measurement of TRAb is useful both as a diagnostic and prognostic tool and can help inform shared decision making regarding the optimal therapy choice in Graves' thyrotoxicosis. High TRAbs suggest a greater likelihood of recurrence but an individual may value the opportunity to avoid long‐term medication despite this; these data suggest that chance is approximately 50% in those who complete a course of ATD.

## Conflicts of Interest

The authors declare no conflicts of interest.

## Supporting information

Thionamide manuscript Clin endo SM.
