# Supplementary material for: Ten Year Outcome of Anti‐Thyroid Drug Treatment for First Episode Graves' Thyrotoxicosis: The Predictive Importance of TRAb
Source: Clin Endocrinol (Oxf). 2025 Jul 8;103(4):612–8. doi: 10.1111/cen.70003 (PMC12413677; doi:10.1111/cen.70003)
Supplement: Supplementary file 1 — Thionamide manuscript Clin endo SM. [file CEN-103-612-s001.docx]

**Supplemental material**

**Supplemental table:** Comparison of demographic and clinical features in people not on thyroid medication at final follow-up and those on either ATD or levothyroxine (median 141 months [minimum 120 months]). Excludes 8 individuals who died after initial 10-year follow-up period.

|  | Not on medication at final follow-up  (n = 94) | On either levothyroxine or ATD at final follow-up (n = 89) | P |
| --- | --- | --- | --- |
| Age at diagnosis (years) | 46 (38 – 56) | 41 (33 – 50) | 0.054 |
| Gender | Female 107 (80%)  Male 26 (20%) | Female 86 (77%)  Male 17 (23%) | 0.560 |
| Free T4 (pM) at diagnosis | 31 (24 – 38) | 32 (25 – 40) | 0.508 |
| Total T3 (nM) at diagnosis | 4.6 (3.5 – 5.7) | 4.8 (3.1 – 5.9) | 0.885 |
| TRAb (IU/L) at diagnosis | 7.4 (4.1 – 11.5) | 7.4 (4.5 – 13.1) | 0.631 |
| Anti-TPO (IU/L) at diagnosis | 83 (23 – 272) | 85 (25 – 389) | 0.643 |
| Duration of therapy (months) | 17 (16 – 18) | 18 (17 – 20) | 0.007 |
| TRAb at cessation (IU/L) | 1.0 (<0.9 – 1.5) | 1.3 (<0.9 – 2.1) | 0.032 |
| Duration of normal TSH (months) | 12 (8 – 15) | 13 (9 – 16) | 0.144 |
| Duration of normal fT4 (months) | 16 (13 – 18) | 16 (14 – 18) | 0.484 |
| Time to normalisation of TSH (months) | 4 (2 – 7) | 4 (3 – 9) | 0.339 |
| Time to normalisation of fT4 (months) | 1 (1 – 2) | 2 (1 – 2) | 0.005 |
